# Supplementary material for: Genetic Dissection of Panicle Morphology Traits in Super High-Yield Hybrid Rice Chaoyou 1000
Source: Plants (Basel). 2024 Jan 9;13(2):179. doi: 10.3390/plants13020179 (PMC10818613; doi:10.3390/plants13020179)
Supplement: Supplementary file 1 [file plants-13-00179-s001.zip › plants-2742015-supplementary.pdf]

Supplemental Table S1: Statistical descriptions of yield related traits in two population derived from Guangxiang 24S and R900

	Trait	Parents		population			
		Guangxiang24S	R900				
		Mean±SD	Mean±SD	Mean±SD	Range	Kurtosis	Skewness
F_2	PN	7.00±1.56	4.00±0.71	4.34±1.24	2.00-11.00	0.85	0.68
	PL	18.07±0.83 cm	22.04±0.76 cm	20.26±1.48 cm	16.60-24.45 cm	-0.13	0.02
	PBN	11.33±0.516	17.80±2.28	15.32±1.58	12.00-20.00	-0.26	0.18
	SBN	27.67±3.011	69.20±17.23	48.43±11.36	26.50-86.00	0.73	0.64
	GN	157.17±13.32	290.20±51.85	253.94±56.50	118.50-403.00	-0.01	0.43
	FGN	132.50±12.62	267.80±58.18	237.99±56.18	108.00-395.00	0.21	0.42
	EGN	24.67±5.495	22.40±20.69	27.49±11.18	14.5-56.00	1.05	1.26
	SSR	84.2%±3.2%	92%±8.1%	94.02%±4.32%	80%-99%	0.32	-1.16
	PSD	8.71±0.73 per cm	13.21±2.56 per cm	12.50±2.45 per cm	5.23-19.44 per cm	0.50	0.48
F_3	PN	9.25±3.49	5.92±2.16	8.89±2.15	5.470-20.250	0.46	0.72
	PL	23.90±0.94 cm	26.13±0.48 cm	27.04±1.84 cm	22.05-29.80 cm	-0.12	0.19
	PBN	12.25±0.96	18.50±1.29	15.90±1.53	12.25-20.00	-0.28	0.43
	SBN	50.25±5.97	84.50±6.56	73.38±12.59	39.00-113.33	0.16	0.60
	GN	235.00±35.64	390.25±26.09	347.07±60.92	209.67-532.00	0.02	0.53
	FGN	15.4±4.98	258.50±25.96	245.72±58.38	42.67-398.75	0.60	0.28
	EGN	182.75±47.97	131.75±25.90	101.36±57.99	13.00-395.00	0.88	0.83
	SSR	6.78%±2.76%	66.28%±4.80%	72.42%±11.62%	31%-94%	-0.02	0.46
	PSD	9.82±1.10 per cm	14.93±0.68 per cm	12.86±2.22 per cm	8.25-19.81 per cm	0.32	-0.64

SD: standard deviation; Trait: PL: panicle length; PN: panicle number per plant; PBN: primary branch number; SBN: secondary branch number; GN:grain number per panicle; FGN: filled grain number per panicle; EGN: empty grain number per panicle; SSR: seed setting rate; PSD: panicle setting density;

Supplemental Table S2: The physical positions and primer information of marker pairs

Chr.	Marker Name	Physical Position (bp)	Primer Information	
			Forward Primer	Reverse Primer
1	RM3252	319100	GGTAACTTTGTTCCCATGCC	GGTCAATCATGCATGCAAGC
	RM6786	7121400	TTGACGATGGCAAGCTCC	GATTTCCGCAGCAAGTATCC
	RM583	8420150	AGATCCATCCCTGTGGAGAG	GCGAACTCGCGTTGTAATC
	RM493	12557603	TAGCTCCAACAGGATCGACC	GTACGTAAACGCGGAAGGTG
	STS	15282195	ATGCTCTGTTTGGCTTATTTCACG	ATACAGTTCGTCCGCTACAGGC
	CM1-6	20278362	GCCACTGCCAGGATCTGTAA	TCAAACATACTCGGTACTTCTCA
	CM1-9	26119472	ATCCTTTGTCCCTCGTCGTC	TGTCCGTCCACTGATCATGT
	CM1-10	30289346	CTTGAGACGTGCTGGTGTG	TCACTGGATCCACATGCCAT
	RM8097	32464694	TACATACACGTTCATGTGCC	CGAGCGTAGGAAGACTACC
	CM1-19	34129535	CGGATGGTCAAACGTTGGAT	AAAGAGTCCCCAGTTCCCG
	CM1-31	40159465	AGCCTCTCCAAATCGAACTCT	ACATGTGGGTTCCTGTGTCA
	STS	43348400	GGGTCATATTTCTATTAATTC	AAGGAAAATCCACCACTAATC
	RM6321	44042256	GGCTCTACCTCGCTGTTGTC	ACGAATATAACCTGCGGCAG
2	CM2-2	54737	TTTTGGCCATGCAAACTCCA	TGCTACACATAGATCCCTCGT
	CM2-3	2166682	TCCGAAAACCCCTTCTGACA	TGGGAGAGAGTGATGGAGGA
	CM2-5	3964175	GGAAACCAGGGACAACGAAC	GGGAACGTGTGTGTGAACAA
	CM2-35	21342166	GCTAAGAATCGAACGGGTCAC	ATGTGTTGAATCGAGCCGTC
	CM2-12	25293066	TCTCGTCGGTAATCTCGTGA	ACAAGATACGACATCAAGCACA
	CM2-13	28641860	AGCTCTTCAAAAGGCAAGTCA	CCGATGCATGACTTCCTTCTG
	RM3763	29600800	TCTCTGAACACACCCACACC	TGTTTTGATCTCAGCTCCCC

	STS	30538814	CCACAACACCAACGATGCTA	TAGGACTGACCCCTCTGCAT
	STS	30925200	GCATAGTTGAACCAGTAGCATG	GAACTGGCAGAATTGAGTGTC
	RM3316	32365700	TTCGACGATTCTGTACACGC	CATGATCCCAAATGCATGGG
	CM2-37	35703922	TCTACCACGTGCCAAAATACA	GTCCATGATCCATCCAAGCAC
	CM2-15	37682703	TGAATCATGGTATGGAGGCAC	AGATCACTGATGGCACGTCA
3	CM3-25	2151293	TCAGATCATCCAGCACAGCA	ATGAGGTAGAAGGGGCCAAC
	CM3-23	5666302	GCAAGAATTAGACAGGAGTAGGT	GTGAGTTTCTACGTTGTGCCA
	CM3-8	13108039	ACTCCTTGCACACATCAAACA	TTATTGTGCTTATAAGGGCTGCA
	STS	16589300	TGTTGTTTTGGAGATTTGAAGG	GAGGCGAAAAGTCACGTAAGC
	CM3-11	18592767	CAAGCTAGCTGGAGGAGGAG	AGATTTCTCCTCCCCAACCG
	CM3-13	25808801	TGCATTTCCCAATTCTGAACCT	CCTGTAGGTTGACTGTTGCA
	CM3-16	31250982	GCATTCATGTCTCTTTAGGGTGT	GGCTCACCTCGTTTTACATATCT
	CM3-18	34893973	GGCCTCTATATCCGCAAGCA	ATCTCGACTTCCTGCTCACC
4	STS	352800	AGAATAGAGTGCATCATCGTC	AACCTGATAGGTGGAAGATGTAC
	CM4-7	3945262	GCCAATGAGATGAAGGACCC	ACCAATGTGTGAAGAAGGAGAT
	CM4-32	9460741	CGGATTCAAACAGGGCCATT	AATTAGCGCGGAATTGACGG
	CM4-15	12469155	CAGTCACAGGCGATCAACAT	TGCAGGACCGTGTAATCAAC
	CM4-34	16820391	TAAAACCCTCGTCCTTCCCC	TCAGCTGGCCACTAAAATGA
	CM4-19	19938120	TGGTAGTTGTGTCTCATGTGC	AACAGGTGTCATGAGCTCCA
	CM4-21	22025422	TCTGTTAGCATCCCTCCACT	TCCCATGCATGCGACAAATT
	CM4-22	23799267	GTCCCCGATATCTTGCAGCT	GCTGACGTGGCTTATTGACT
	RM3839	24155150	AATGGGACCAGAAAGCACAC	AAAAAGAGCATGGGGGCTAC

	CM4-25	27307764	TTGGCGGCTTGGTGTAGT	AAATGCCGCCATCTATACAGT
	CM4-27	30155206	TAGGTATGGATTGAGTGGGTCT	TTCGACTGAATGGGAATATGCT
	RM349	32971050	TTGCCATTCGCGTGGAGGCG	GTCCATCATCCCTATGGTCG
	RM1113	34535150	GGGCGCATGTGTATTTCTTC	TGGGGAAAAACCACAAGCC
	CM4-29	35372209	TGAAACATGAAACAAATCCGAGT	TGAAACAGCGCCCGATTG
5	CM5-1	1969011	GATGCCGGACAAGCTTGAAT	TGTTCCAAACTCCAAACATGCA
	CM5-4	3518668	ACGAATGGTCAAACATGTGAGA	TGGAGTACTGTACGTTCGCT
	CM5-5	6165831	GTCTCTTTTGCACCGTGACC	GCAAGGGCGTTAGTAGAATTTT
	CM5-12	18983377	TGCCAATTGTAGGTGTTGTGT	CACTTGATGGGATTGCTTGCT
	CM5-14	21885780	AAATCCGTGGCCCTCCTC	ATAGGGGAGGGAGTGGTGAT
	CM5-16	25003997	ACATGGAGTAGCAATCGACCA	TGCCTGTGGTTTTCGTTACG
	CM5-17	27701004	GTGCGGATTTCTCCTCTCTC	TGCCGGTCCATTTCTAGAAG
	CM5-19	28837876	TTGAAACCCCAGTAGCAGCT	AGCTGAGAGAGAAGAGGGGA
	CM5-20	30147722	CAAGCCAGAGTCGACGATTC	TGCCACTCCAATCTCTCTCTC
	CM5-21	31069576	GGAGGGGTGGCCATGTATTA	GCCGGCTTGCTGACTATTAG
6	CM6-18	50357	CGGCCATGGTAGCTAGTGTA	CCCGAGCTCAGATCATTGGA
	CM6-19	2058467	ATGCAATGTCAGGCGAAACT	TGGTGGTTGAAATTGTTGCAG
	RM217	4059800	ATCGCAGCAATGCCTCGTG	TGCGTTTGTGTTTGGCTCG
	RM19715	7947000	CCAATCTAAATTACGCCGCTAGG	GCCAGCTGTTGTTTGTAGTTTCG
	CM6-21	10486154	CATACGTGCCTGAGTTTCCG	TCTCTCTTTCCTGCCCTGTT
	CM6-11	14064647	ATCCTGATCCTTCCTTGCCG	TCGCCTACTCAATCGACGG
	CM6-13	15772370	TGGCCCAGCAACTATCGAG	ATGTACCAAGAGCTTCGCCT

	CM6-14	17528519	CTTTTATCTCCATGGCCGCC	CTACTGTCGCACCTCCTTCC
	CM6-26	20558014	CACGCTGTGTCTTCCGTTAA	ATTGCTGTAGAGGCCTGGTT
	CM6-28	22384871	TAATTCACCACTTCCACCGC	AGTGTTGTGATTTGCAGGGC
7	STS	230388	TATGTCCTTAGCATGGAAGACC	CGTTGTTGATCATCTGGTACG
	CM7-2	4570930	TGAGGCTTGACTTGGATGGA	CAAACGAAACGAACGCCAAC
	STS	6071141	TTCTTCCATGTAGCAAGCATT	CCTACTGCCTGCCAAATCTAT
	CM7-10	8013115	CACATGGACAAAACAAAGGCA	TGAACGGTCATGTATGGTTTGA
	RM3755	14833204	TGTGGACAACCTCAACTGAAAGC	CATAATCACCAACATCGGAGAAGC
	CM7-6	16779869	AGCAAGCCGTCCTGTTCTT	CCGGTGATTCGTCGTCAG
	RM11	19866268	TCTCCTCTTCCCCCGATC	ATAGCGGGCGAGGCTTAG
	RM1132	24658736	TCAAGGTCGACATGTTAGGTATGC	AACCCTATCACCTGAGAAACATCC
	RM3555	28314588	TGGAAGTTTCCTGGCGATAG	TGGTTGGACTGAAAAGTCCC
8	RM5556	4531170	ATCTCCCTCCCTCTCCTCAC	TCCACACCTTCACAGTTGAC
	CM8-6	7674992	TTTAGACTTTGGTTGTGGCTAAC	TGGCTCATACTCTCAGACCA
	CM8-19	9392969	TCCCGGCGTCATATCTCTTC	GACTTGTATCGCCCAACACC
	CM8-11	12800306	GGCGAAGAAAACCCGGTG	GTCGAGTTTTGATCTCCGGC
	CM8-13	14440957	ACCACTACTGTTGAGGGCTC	GCAGCGACAGACTTGGTC
	CM8-21	18439042	CAAACGGGATCATCGGTCG	CGCCTTGTCCGATTCCAATT
	CM8-15	22826955	GGCAACTGTTCTCCAATTTGT	TCTGCCTGTTACTCCACCTT
	CM8-23	24081606	GTAACCGTGTTTACCGCCG	TAATCTCCTCGCGCACTAGG
	CM8-17	28644785	GGGAAGTACAATGTGAGGGC	CCACGTTTTCACCAGTAACCA
9	CM9-1	132368	CCACTTTCCACACTTGAGGC	CGGAGGTCTATGTTGGGTGA
	STS	1800000	CCTGGTTAGCACTACAG	TAGTTGGCTATGTCCACA

			CTC	CC
	CM9-6	5086933	TTTTGGTGTTTCATCCCCGG	TTTCTCCGTTTGACACCTGC
	CM9-7	9141690	AGGTAGCCTCGACATTAACGT	CAGCGTCAATCCATCGATCG
	CM9-9	12219870	TCCTAGGTGATGTGGTGTTGT	AGGGGCGAGCATAAGGTAAA
	CM9-10	13261506	GAAGGCGTCATCCATTTCCG	AGATGCAGGAGAGGATGAGC
	CM9-13	17254623	TCACAACTCACAAGCCATGC	TCGGTCTCTATCTCGGTCTTT
	CM9-15	19117217	ACACCATGAGTACACCAACAA	AGCCTACTCACGGACTGTTT
	STS	22641750	TGACTCAAGGCATATGCAGC	TTGTCCATGCTGTTAGCCTG
10	STS	1100000	GCATAGCGTGACCACCGAGAA	ACCGCACAATGATTGGGAAATAC
	CM10-4	4321523	AATTCCTGAGCAGAAAGTTTGTT	CATTTCGAACACTAGGGTCTGT
	CM10-7	6023938	AGAGCTTCCATCTGTACAGC	GGTTTGGAATATTCATGAGCCCT
	CM10-9	11676474	CATCACCTCACCCTACCCG	CGGTGAACTCGGTCCACT
	CM10-10	13332668	ACCTCCAAACCAAACACGTT	TGGAAGGGCTTGTTCACTTG
	RM5689	15482350	GCACATGGTGAGACGTCCTC	AAGTCCTGTAGTAGGTCACACCG
	RM1375	18668850	CTACACGCGCAAACTCTGTC	ATGAAGGTCTAGGCTGCACC
	CM10-14	21113520	TGCTCCTTACCACCAACCAT	TCTACCAGTACCGCACCAAG
	CM10-16	24181832	CTCTAGGAGGAGTGGAGCTG	CTACTCATTCCGTTTCACAATGT
11	CM11-5	5211088	TTTCGTCTTCAAAAGGGTTTTCA	GGAAGAAGATGAAAATGACCAGG
	STS	7903900	CGCTTGAAAGGACTCCAGAC	CCATCTACTCACCAAACGTTCC
	CM11-7	9163957	GGTCTGCTTAGATCCAAAGGC	GGAACTGACTTAGAATGGACAGC
	CM11-12	12265617	AAGCTTTAGGAGTTAGTCGGTT	CTGTCTGGCCCTGAACAAAC
	CM11-32	13710632	AAGATGGTGACGAACGGACA	AATGGGCCCAACTATCATGC
	CM11-39	15054692	GTTCTTTCCTCTTGCCCG	ATCAACTGAGTTTGCTGG

			TG	GC
	CM11-41	17212175	ATCAGGACAGATGCCCTTCA	AAGTCCAGCCTCATCACGAT
	CM11-21	22085028	CCCAAATCACACACTCTTCGT	CCGCTGCTCTTATCTCTCCT
	CM11-29	24203520	CGTCTTGTTAGCTGCCGAAT	GGCTTCTAAAACGGTGCTCC
	CM11-23	25500867	GGGCAAATAAACTGTGTCACC	CAAGGGGAAAACGGCCATC
	CM11-37	2966139	GCAGGCATGGACAAACTTCA	CTGATGAGTTGGCATATGTCCC
12	CM12-4	3363990	ATGGACCATGGCTCAGCAG	GCACCGCAGCACCAGATG
	CM12-6	6061157	GCCCAGCTACAAATACCCCA	TGGTTGTCTTTCCTTATCCGGA
	RM101	7782800	GTGAATGGTCAAGTGACTTAGGTGGC	ACACAACATGTTCCCTCCCATGC
	RM1337	11058050	GTATCCTTTCTCGCAATCGC	GTGGCCCGAACCTGTATAAC
	CM12-11	14353137	ATTTCCGCCACAGATTTGCC	GGGGAAGAGATTGAGCCAGA
	CM12-15	18412824	AGTTGAGGTATAAGGCCCCG	TGGCCATGTCACTGCTGATA
	SSR	20654550	GGAGTGGAAGACATCAGGGA	AGAAGGCTCGATTGTTGCAT
	CM12-27	23723600	CACTTTGTGTCGTGCTTGGG	CTAGCCTAGACACGAGACGG
	CM12-28	25594702	ATCACCGTTTGCATTCAGGC	CGTTTGCCCTGTTTGTTTGT
